# Is the effect of Mediterranean diet on hip fracture mediated through type 2 diabetes mellitus and body mass index?

**DOI:** 10.1093/ije/dyaa239

**Published:** 2020-12-25

**Authors:** Adam Mitchell, Tove Fall, Håkan Melhus, Alicja Wolk, Karl Michaëlsson, Liisa Byberg

**Affiliations:** 1 Department of Surgical Sciences, Orthopaedics, Uppsala University, Uppsala, Sweden; 2 Department of Medical Sciences, Molecular Epidemiology, Uppsala University, Uppsala, Sweden; 3 Department of Medical Sciences, Clinical Pharmacogenomics and Osteoporosis, Uppsala University, Uppsala, Sweden; 4 Institute of Environmental Medicine, Cardiovascular and Nutritional Epidemiology, Karolinska Institutet, Stockholm, Sweden

**Keywords:** Causality, Mediterranean diet, hip fractures, type 2 diabetes mellitus, body mass index

## Abstract

**Background:**

We examined whether the inverse association between adherence to a Mediterranean diet and hip fracture risk is mediated by incident type 2 diabetes mellitus (T2DM) and body mass index (BMI).

**Methods:**

We included 50 755 men and women from the Cohort of Swedish Men and the Swedish Mammography Cohort who answered lifestyle and medical questionnaires in 1997 and 2008 (used for calculation of the Mediterranean diet score 9mMED; low, medium, high) and BMI in 1997, and incident T2DM in 1997–2008). The cumulative incidence of hip fracture from the National Patient Register (2009–14) was considered as outcome.

**Results:**

We present conditional odds ratios (OR) 9[95% confidence interval, CI) of hip fracture for medium and high adherence to mMED, compared with low adherence. The total effect ORs were 0.82 (0.71, 0.95) and 0.75 (0.62, 0.91), respectively. The controlled direct effect of mMED on hip fracture (not mediated by T2DM, considering BMI as an exposure-induced confounder), calculated using inverse probability weighting of marginal structural models, rendered ORs of 0.82 (0.72, 0.95) and 0.73 (0.60, 0.88), respectively. The natural direct effect ORs (not mediated by BMI or T2DM, calculated using flexible mediation analysis) were 0.82 (0.71, 0.95) and 0.74(0.61, 0.89), respectively. The path-specific indirect and partial indirect natural effects ORs (through BMI or T2DM) were close to 1.

**Conclusions:**

Mediterranean diet has a direct effect on hip fracture risk via pathways other than through T2DM and BMI. We cannot exclude mediating effects of T2DM or BMI, or that their effects cancel each other out.

**Key Messages**

- Application of recently developed statistical methods for mediation analysis may provide insights into the underlying mechanisms of the effect of Mediterranean diet on the risk of hip fracture.
- Mediterranean diet has an effect on hip fracture risk directly or via pathways not including type 2 diabetes mellitus (T2DM) or body mass index (BMI).
- Other pathways than via T2DM and BMI should be explored for further understanding of how adherence to a Mediterranean diet would be beneficial for hip fracture risk.

## Introduction

Hip fracture is the most devastating frailty fracture in the elderly population,^1^ associated with severe consequences for health and quality of life,^2^ high health care costs^3^ and increased mortality.^4^ A diet rich in vegetables such as the ‘Mediterranean diet’ is associated with lower risk of hip fracture in both men and women.^5^^,^^6^ The underlying mechanisms are largely unknown and have not been investigated using mediation analysis.

**Figure 1:**
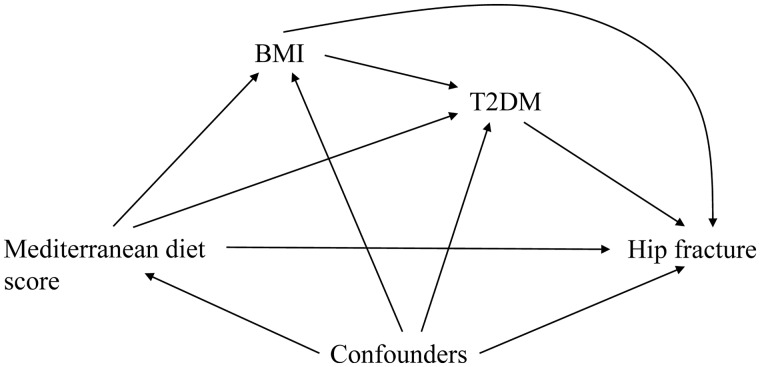
Causal diagram for the hypothesized effects of Mediterranean diet on fracture risk via body mass index (BMI) and type 2 diabetes (T2DM) in the presence of baseline confounders (C)

Adherence to a Mediterranean-style dietary pattern is associated with a reduced risk of type 2 diabetes mellitus (T2DM),^7–9^ which itself is associated with an increased risk of hip fracture.^10^^,^^11^ This could be a potential mechanistic pathway for the effect of Mediterranean diet on hip fracture risk. Another variable to consider in this complex relationship is body mass index (BMI). The majority of evidence suggests that a Mediterranean diet lowers BMI.^12^ At the same time, BMI is associated with greater risk of T2DM,^13^ whereas BMI is in general inversely associated with fracture risk^14^ although it might be possible that those in the highest BMI range also have an increased risk of fracture at certain sites.^15^ The assumed directions of the causal effects are illustrated in Figure 1. Throughout, we assume that the causal assumptions underlying this causal diagram hold.

Mediation analysis aims to separate indirect effects acting through one or more mediators from the remaining direct effect.^16^ Mediation analysis using standard regression methods may provide biased estimates in the presence of exposure-induced mediator-outcome confounders,^17^^,^^18^ both when omitted (residual confounding) and when included (blocking other mediating paths) in the regression model. Modern mediation analysis uses explicit counterfactual scenarios and allows for decomposition of effects when conventional approaches are biased.^18^

The main purpose of this study was to estimate the controlled direct effect of self-reported adherence to a Mediterranean diet (in 1997) on the risk of hip fracture (in 2009–14) which is not mediated through incident T2DM (in 1997–2009). In addition to regression models where we condition on T2DM as a mediator, we applied inverse probability weighting of marginal structural models (MSM) considering BMI as an exposure-induced confounder of the T2DM-hip fracture association,^19^^,^^20^ to estimate this effect. In view of the known effects of BMI on T2DM, we further aimed to explore different mediating paths and therefore applied flexible mediation analysis to estimate the natural direct, indirect and partial indirect effects, where BMI and T2DM are regarded as multiple causally ordered mediators.^21^ We discuss the results from an applied user perspective without going deep into statistical theory.

## Methods

The research was performed in accordance with the Declaration of Helsinki and was approved by the regional ethics review boards at Uppsala University, Uppsala, Sweden, and Karolinska Institutet, Stockholm, Sweden. All participants gave their informed consent.

### Study population

We included 50 755 participants with information on exposure in 1997 and mediator in 2008 from two population-based cohorts based in central Sweden: the Swedish Mammography Cohort (SMC) and the Cohort of Swedish Men (COSM) (Supplementary Figure S1, available as Supplementary data at *IJE* online), both components of the national research infrastructure SIMPLER [www.simpler4health.se]. Participants responded to questionnaires that included information on height, weight, diet, alcohol consumption, diabetes status, education and living conditions, smoking status, physical activity and other lifestyle factors in 1997 and in 2008. At baseline in 1997, we excluded participants with cancer (except non-malignant skin cancer) or diabetes.

### Hip fracture (outcome)

We linked each participant with the National Patient Register for identification of incident hip fractures [International Classification of Diseases (ICD)-10 codes S720, S721 or S722]. The register covers all inpatient care in Sweden since 1987 and is valid for hip fracture identification^22–24^ with minimal loss to follow-up. The cumulative incidence of hip fracture between 14 April 2009 and 31 December 2014 was used as outcome (shown by age and sex in Supplementary Figure S2, available as Supplementary data at *IJE* online). We used a valid method to distinguish incident hip fractures from readmissions of previous hip fractures.^25^

### Mediterranean diet score (exposure)

The modified Mediterranean diet score (mMED; range 0–8 points) represents relative adherence to a traditional Mediterranean dietary pattern^26–28^ and was calculated from a food frequency questionnaire in 1997 (Supplementary Methods, available as Supplementary data at *IJE* online). One point was given for intakes above the median for: fruit and vegetables; legumes and nuts; non-refined or high-fibre grains; fermented dairy products; and fish. Further, one point was given for: intakes below the median of red and processed meat; use of olive or rapeseed oil for cooking or as dressing; and moderate alcohol consumption (5–15 g ethanol/day).

### Mediator(s)

At baseline (1997) participants were free from diabetes; thereby we defined T2DM as incident diabetes using self-reported diabetes diagnoses from the 2008 questionnaire, which has been shown to be a valid form of disease definition.^29^ As mean age at baseline 1997 was 59 years, we assumed the majority of incident diabetes cases to be T2DM. BMI at baseline (1997) was calculated as weight (kg) divided by the height (m) squared.

### Confounders

Measured confounders of mMED and hip fracture risk included at baseline: age and sex; variables collected from questionnaires: educational level (primary school, high school, university), physical activity (five categories), smoking status (current, former, never), living alone status [yes (unmarried, divorced, widows/widowers), no (married or cohabiting)], calcium supplement use (yes, no), vitamin D supplement use (yes, no), total energy intake (kcal/day); and based on inpatient treated diseases from the National Patient Register before 1 January 1998: Charlson’s weighted comorbidity index^30^ which was modified to not include diabetes. Educational level was used as a marker of socioeconomic status.

### Statistical analyses

To reduce the number of parametric assumptions needed when modelling the exposure,^19^^,^^21^ we categorized the mMED into three predetermined categories: 0–2 (low), 3–5 (medium) and 6–8 points (high).

Based on Figure 1 and using the annotation in Table 1, we estimated the total effect of mMED (A) in 1997 on hip fractures occurring 2009–14 (Y) using logistic regression adjusting for baseline confounders (C) (Table 1, Model 1). We subsequently applied three different methods to evaluate the conditional controlled direct effect not mediated by T2DM and to evaluate different mediating paths, treating BMI and T2DM as causally ordered mediators (see below and Supplementary Methods). The same set of baseline confounders (C) were used in all three applications. For all models, we performed 20 imputations of missing data on covariates using multiple imputations with chained equations and calculated pooled estimates and 95% confidence intervals (CI) using Rubin’s rules. The proportion of missing data was no more than 10%.

**Table 1:** The statistical models used to determine the total effect, controlled direct effects and natural direct and indirect effects

Model	Counterfactual notation and estimation model	Effect description and effect paths^a^
Total effect of mMED on hip fracture risk		
Model 1	$\frac{E\{Y(1)\;|\;C\}\;/\;(1-E\;\{Y(1)|C\})}{E\{Y(0)\;|\;C\}\;/\;(1-E\;\{\;Y(0)|C\})\;}$ Estimated by: $\text{logit}\;\hat{P}\left(Y_{i}=1|A_{i}=a,\;C_{i}=c\right)={\hat{\beta }}_{0}+{\hat{\beta }}_{1}A_{i}+{\hat{\beta }}_{2}C_{i}$	exp($\hat{\beta }$_1_) corresponds to (the population parameter) exp(β_1_), which corresponds to the target estimand; the total effect odds ratio of mMED (A) on hip fracture (Y) given that measured confounders (C) suffice to control for confounding. The effect paths include: $\begin{array}{c} \text{mMED}\to \text{hip}\;\text{fracture}\\ \text{mMED}\to T2\text{DM}\to \text{hip}\;\text{fracture}\\ \text{mMED}\to \text{BMI}\to \text{hip}\;\text{fracture}\\ \text{mMED}\to \text{BMI}\to T2\text{DM}\to \text{hip}\;\text{fracture} \end{array}$
The conditional controlled direct effect of mMED on hip fracture risk with respect to T2DM as a mediator $\frac{E\{Y(1,\;m)\;|\;C\}\;/\;(1-E\{Y(1,\;m)\;|\;C\})}{E\{Y(0,\;m)\;|\;C\}\;/\;(1-E\{Y(0,\;m)\;|\;C\})}\;$		
Regression methods		
Model 2	Estimated by: $\text{logit}\;\;\hat{P}\left(Y_{i}=1|A_{i}=a,\;M_{i}=m,\;C_{i}=c\right)={\hat{\gamma }}_{0}+{\hat{\gamma }}_{1}A_{i}+{\hat{\gamma }}_{2}M_{i}+{\hat{\gamma }}_{3}C_{i}$	exp($\hat{\gamma }$_1_) corresponds to (the population parameter) exp(γ_1_), which corresponds to the target estimand; the conditional controlled direct effect odds ratio of mMED (A) on hip fracture (Y), not via T2DM (M), given the assumptions that measured baseline confounders (C) suffice to control for confounding between: (i) mMED and hip fracture; (ii) T2DM and hip fracture; and (iii) that there is no exposure-induced confounding. The effect paths include: $\begin{array}{c} \text{mMED}\to \text{hip}\;\text{fracture}\\ \text{mMED}\to \text{BMI}\to \text{hip}\;\text{fracture} \end{array}$ Under the causal DAG in Fig 1, one may expect exp($\hat{\gamma }$_1_) to be biased^b^ due to residual confounding from the exposure-induced mediator-outcome confounder BMI through the biasing path mMED → T2DM ← BMI → hip fracture, which is opened upon conditioning on T2DM
Model 3	Estimated by: $\text{logit}\;\;\hat{P}\left(Y_{i}=1|A_{i}=a,\;M_{i}=m,\;C_{i}=c,\;L_{i}=l\right)={\hat{\delta }}_{0}+{\hat{\delta }}_{1}A_{i}+{\hat{\delta }}_{2}M_{i}+{\hat{\delta }}_{3}C_{i}+{\hat{\delta }}_{4}L_{i}$	exp($\hat{\delta }$_1_) corresponds to (the population parameter) exp(δ_1_), which corresponds to the target estimand; the conditional controlled direct effect odds ratio of mMED (A) on hip fracture (Y), not via T2DM (M), given that measured baseline confounders (C) and BMI (L), suffice to control for confounding between: (i) mMED and hip fracture; (ii) T2DM and hip fracture; and (iii) that there is no exposure-induced confounding. The effect path includes: mMED→hip fracture exp(δ_1_) may suffer from bias^b^ since conditioning on BMI blocks one of the pathways of interest (mMED →BMI → hip fracture). If assumption (iii) is violated and there is unmeasured confounding (U) between BMI and hip fracture, exp(δ_1_) may further suffer from collider bias through the biasing path mMED → T2DM → BMI ←U → hip fracture. which is opened upon conditioning on BMI Note that exp(δ_1_) is not a biased estimate of the controlled direct effect with respect to T2DM and BMI as a set of joint mediators
Inverse probability weighting of marginal structural models conditional on confounders^c^		
Model 4	Estimated by: $\text{logit}\;\;\hat{P}(Y_{i}=1|A_{i}=a,\;M_{i}=m,\;C_{i}=c)={\hat{\eta }}_{0}+{\hat{\eta }}_{1}A_{i}+{\hat{\eta }}_{2}M_{i}+{\hat{\eta }}_{3}C_{i}$ where each individual is weighted by $w_{M}{}^{d}$	exp($\hat{\eta }$_1_) corresponds to (the population parameter) exp(η_1_), that corresponds to the target estimand; the conditional controlled direct effect odds ratio of mMED (A) on hip fracture (Y) not via T2DM (M), given that measured baseline confounders (C) and BMI (L)suffice to control for confounding between: (i) mMED and hip fracture; and (ii) T2DM and hip fracture. exp($\hat{\eta }$_1_), is estimated by fixing the mediator T2DM (M) to m and controlling for the exposure-induced mediator-outcome confounder BMI through weighting by w_M_. The effect paths include: $\begin{array}{c} \text{mMED}\to \text{hip}\;\text{fracture}\\ \text{mMED}\to \text{BMI}\to \text{hip}\;\text{fracture} \end{array}$ This estimate is not biased even in the presence of unmeasured confounding (U) between BMI and hip fracture.
Natural direct and indirect effects of mMED on hip fracture risk with respect to BMI and T2DM as causally ordered mediators, estimated by flexible mediation analysis		
Model 5 (Estimation model)	Natural effects model = E{Y(a, M_1i_(aʹ), M_2i_(a′′, M_1i_(aʹ)))|C} Estimated by: $\text{logit}\;\;\hat{P}(Ê\;|\;a,\;\mathrm{a'},\;\mathrm{a\prime \prime},\;C)={\hat{\theta }}_{1}a+{\hat{\theta }}_{2}\mathrm{a'}+{\hat{\theta }}_{3}\mathrm{a\prime \prime}+{\hat{\theta }}_{4}C$ where each observation is weighted by $w_{NE}{}^{e}$	The natural effect model decomposes the total effect into three causal pathways corresponding to a (the natural direct effect), aʹ (the natural indirect effect), and a′′ (partial indirect effect), given that measured baseline confounders (C) suffice to control for confounding between: (i) mMED and hip fracture; (ii) the mediators (BMI, T2DM) and hip fracture conditional on mMED; (iii) mMED and mediators (BMI, T2DM); and (iv) that no confounders of the relationship between mediators (BMI, T2DM) and hip fracture are affected by mMED Ê represents the imputed nested counterfactuals based on the fitted values from the outcome model
Natural direct effect with respect to BMI and T2DM as mediators (NDE)	$\frac{E\left\{Y(1,M_{1}\left(aʹ\right),M_{2}\left(a\prime \prime ,M_{1}\left(aʹ\right)\right)\;\right\}/\left(1-E\left\{Y(1,M_{1}\left(aʹ\right),M_{2}\left(a\prime \prime ,M_{1}\left(aʹ\right)\right)\;\right\}\right)}{E\left\{Y(0,M_{1}\left(aʹ\right),M_{2}\left(a\prime \prime ,M_{1}\left(aʹ\right)\right)\;\right\}/\left(1-E\left\{Y(0,M_{1}\left(aʹ\right),M_{2}\left(a\prime \prime ,M_{1}\left(aʹ\right)\right)\;\right\}\right)}$ Estimated by the component $E_{A}{}_{\to Y}(a\prime ,\;a\prime \prime )={\hat{\theta }}_{1}$	exp($\hat{\theta }$_1_) corresponds to (the population parameter) exp(θ_1_), that corresponds to the target estimand; the natural direct effect odds ratio of mMED (A) on risk of hip fracture (Y) through neither BMI (M_1_) nor T2DM (M_2_). The effect path includes: mMED→hip fracture
Natural indirect effect (via BMI)	$\frac{E\left\{Y(1,M_{1}\left(aʹ\right),M_{2}\left(a\prime \prime ,M_{1}\left(aʹ\right)\right)\;\right\}/\left(1-E\left\{Y(1,M_{1}\left(aʹ\right),M_{2}\left(a\prime \prime ,M_{1}\left(aʹ\right)\right)\;\right\}\right)}{E\left\{Y(0,M_{1}\left(aʹ\right),M_{2}\left(a\prime \prime ,M_{1}\left(aʹ\right)\right)\;\right\}/\left(1-E\left\{Y(0,M_{1}\left(aʹ\right),M_{2}\left(a\prime \prime ,M_{1}\left(aʹ\right)\right)\;\right\}\right)}$ Estimated by the component $E_{A}{}_{\to M1\to Y}(a,\;a\prime \prime )={\hat{\theta }}_{2}$	exp($\hat{\theta }$_2_) corresponds to (the population parameter) exp(θ_2_)that corresponds to the target estimand; the natural indirect effects odds ratio mediated by exposure-induced changes in BMI (M_1_). The effect paths include: $\begin{array}{c} \text{mMED}\to \text{BMI}\to \text{hip}\;\text{fracture}\\ \text{mMED}\to \text{BMI}\to T2\text{DM}\to \text{hip}\;\text{fracture} \end{array}$
Partial indirect effect (via T2DM only)	$\frac{E\left\{Y(a,M_{1}\left(aʹ\right),M_{2}\left(1,M_{1}\left(aʹ\right)\right)\;\right\}/\left(1-E\left\{Y(a,M_{1}\left(aʹ\right),M_{2}\left(1,M_{1}\left(aʹ\right)\right)\;\right\}\right)}{E\left\{Y(a,M_{1}\left(aʹ\right),M_{2}\left(0,M_{1}\left(aʹ\right)\right)\;\right\}/\left(1-E\left\{Y(a,M_{1}\left(aʹ\right),M_{2}\left(0,M_{1}\left(aʹ\right)\right)\;\right\}\right)}$ Estimated by the component $E_{A}{}_{\to M2\to Y}(a,\;a\prime )={\hat{\theta }}_{3}$	exp($\hat{\theta }$_3_) corresponds to (the population parameter) exp(θ_3_) that corresponds to the target estimand; the partial indirect effect odds ratio mediated solely by exposure-induced changes in T2DM (M_2_). The effect path includes: mMED→T2DM→hip fracture

BMI, body mass index; C, confounders; mMED, Mediterranean diet score; T2DM, type 2 diabetes mellitus; DAG, directed acyclic graph.

a Effect paths based on the assumed directions of causal effects as illustrated in Figure 1. All models assume positivity and correct model specification.

b For a more elaborate explanation and a causal diagram illustrating the assumptions necessary for an unbiased estimation, please see Supplementary Methods.

c Description of a model estimating marginal controlled direct effects can be found in Supplementary Methods, and the results are presented in Supplementary Table S1.

d $w_{M}$ was calculated based on stabilized weights for the mediator (T2DM), as detailed in Supplementary Methods.

e $w_{NE}$ was calculated based on a model for the mediator (T2DM), as detailed in Supplementary Methods.

### Traditional methods for estimating the conditional controlled direct effect with respect to T2DM as a mediator

To Model 1, estimating the total effect, we added T2DM (*M*) as a covariate in the logistic regression model of the total effect of mMED on fracture risk, conditional on the set of confounders (*C*) (Table 1, Model 2). BMI (*L*) was thereafter included as a covariate (Table 1, Model 3). Both these models may provide biased estimates, assuming the causal effects outlined in Figure 1 (see Table 1 and Supplementary Methods).

### Inverse probability weighting (IPW) of marginal structural models (MSM) for estimating the conditional controlled direct effect with respect to T2DM as a mediator

Based on Figure 1 and assuming no unmeasured confounding, positivity and correct model specification, we estimated the conditional controlled direct effect of mMED on the risk of hip fracture not mediated by T2DM, using IPW of MSM using a weight for the mediator and conditioning on baseline covariates^18–20^ (Table 1, Model 4), as detailed in Supplementary Methods,. This method eliminates the potential bias from the exposure-induced mediator-outcome confounding present in Models 2–3. Robust standard errors using the sandwich estimator were calculated.

### Flexible multiple mediator approach for estimation of natural direct and indirect effects

To further separate the potential mediating paths (Figure 1), we considered BMI as a causally ordered mediator that precedes T2DM, and applied flexible mediation analysis^21^ (Table 1, Model 5; Supplementary Methods). The odds ratios for the three auxiliary variables (a, aʹ and a′′) that are created in the modelling correspond to the causal pathways we wish to decompose: the natural direct effect of mMED on the risk of hip fracture through neither BMI nor T2DM (mMED→hip fracture; a), the natural indirect effect mediated by exposure-induced changes in BMI (mMED→BMI→hip fracture and mMED→BMI→T2DM→hip fracture; aʹ), and the partial indirect effect mediated solely by exposure-induced changes in T2DM (mMED→T2DM→hip fracture; a′′) (Table 2, Model 5). There were no substantial interactions between the auxiliary variables a, aʹ and a′′ used in the natural effects model (Supplementary Methods). Calculation of confidence intervals was performed with 1000 bootstrap samples in each of the 20 imputed datasets (Supplementary Methods).

**Table 2:** Descriptive characteristics of the study population (from Swedish Mammography Cohort and Cohort of Swedish Men combined) by category of adherence to the Mediterranean diet score

	Mediterranean diet score (mMED)			
	Low adherence 0–2 points	Medium adherence 3–5 points	High adherence 6–8 points	Total
Total number (%)	9284 (18.3)	31 830 (62.7)	9641 (19.0)	50 755
Sex				
Female	4132 (44.5)	14 263 (44.8)	5016 (52.0)	23 411 (46.1)
Male	5152 (55.5)	17 567 (55.2)	4625 (48.0)	27 344 (54.0)
Age (years), mean (SD)	59.66 (8.6)	58.99 (8.3)	58.62 (7.9)	59.04 (8.3)
Height (cm), mean (SD)	171.1 (9.0) *n* = 9017	172.0 (8.9) *n *= 31 191	171.7 (8.8) *n* = 9519	171.8 (8.9)
Body mass index (kg/m^2^), mean (SD)	25.6 (3.7) *n* = 8938	25.4 (3.4) *n* = 31 007	24.9 (3.3) *n *= 9481	25.3 (3.5) *n *= 49 426
Energy intake (kcal), mean (SD)	1902 (734)	2303 (840)	2539 (827)	2274 (843), n = 47 477
Live alone				
Yes	1781 (20.5)	5008 (16.8)	1314 (14.7)	8103 (17.1), *n* = 46 172
Physical activity				
<1 h/week	2247 (27.2)	5781 (19.9)	1187 (13.3)	9215 (20.0)
1 h	1883 (22.8)	6503 (22.4)	1886 (21.2)	10 272 (22.3)
2-3 h	2442 (29.6)	9695 (33.4)	3261 (36.6)	15 398 (33.4)
4-5 h	840 (10.2)	3510 (12.1)	1254 (14.1)	5604 (12.1)
>5 h/week	847 (10.3)	3517 (12.1)	1319 (14.8)	5683 (12.3), *n* = 50 083
Smoking status				
Never	2400 (26.3)	6995 (22.3)	1723 (18.0)	11 118 (22.2)
Previous	2680 (29.3)	10 230 (32.6)	3403 (35.6)	16 313 (32.6)
Current	4059 (44.4)	14 167 (45.1)	4426 (46.3)	22 652 (45.2), *n* = 50 689
Education				
Primary school	7094 (76.6)	21 209 (66.7)	5360 (55.6)	33 663 (66.4)
High school	978 (10.6)	3948 (12.4)	1369 (14.2)	6295 (12.4)
University	1170 (12.6)	6552 (20.6)	2887 (30.0)	10 609 (20.9)
Vocational	24 (0.3)	79 (0.3)	19 (0.2)	122 (0.2)
Vitamin D supplement use	1373 (14.8)	6164 (19.4)	2426 (25.2)	9963 (19.6)
Calcium supplement use	1477 (15.9)	6610 (20.8)	2627 (27.3)	10 714 (21.1)
Charlson comorbidity index^a^				
0	8905 (95.9)	30 774 (96.7)	9315 (96.6)	48 994 (96.5)
1	289 (3.1)	783 (2.5)	236 (2.5)	1308 (2.6)
≥2	90 (1.0)	273 (0.8)	90 (0.8)	453 (0.9)
Incident type 2 diabetes (1997-2008)^b^	717 (7.7)	2134 (6.7)	538 (5.6)	3389 (6.7)
Hip fracture (2009-14)	322 (3.5)	841 (2.6)	223 (2.3)	1386 (2.7)

a Charlson comorbidity does not include diabetes.

b Incident type 2 diabetes (between 1997 and 2008) from self-reported questionnaire.

All statistical analyses were performed using resources provided by SNIC-SENS through the Uppsala Multidisciplinary Center for Advanced Computational Science (UPPMAX), using StataMP 15 (Stata Corp., Collage Station, TX, USA).

## Results

Characteristics of the study population are shown in Table 2. Those with the highest adherence to the Mediterranean diet were more likely to be: female (52.0% vs 44.5%); not living alone; more physically active; and to have a higher attained educational level and more frequently take supplements containing calcium and vitamin D. The incidence of T2DM in 1997–2008 (6.7%) was highest in those with the lowest adherence to Mediterranean diet. In the 6-year follow-up period, 1386 (2.7%) men and women suffered a hip fracture.

The total effect model indicated that those in the second level and with highest adherence to mMED had, respectively, 18% {odds ratio [OR] = 0.82 [95% confidence interval (CI) = 0.71, 0.95]} and 25% [0.75 (0.62, 0.91)] lower odds of hip fracture compared with those in the lowest adherence category (Table 3, Model 1). The potentially biased conditional controlled direct effect ORs of mMED with respect to T2DM as a mediator on hip fracture risk estimated using traditional methods including T2DM in Model 2, and including both T2DM and BMI in Model 3, were similar to the total effect ORs. The conditional controlled direct effects ORs of mMED on hip fracture risk with respect to T2DM as a mediator estimated using IPW of MSM (Model 4) were 0.82 (0.71, 0.95) and 0.73 (0.60, 0.88) for medium and high adherence compared with low mMED adherence. Whereas all these estimates aim to measure the conditional controlled direct effect of mMED on the risk of hip fracture not through T2DM, the latter estimates appropriately control for BMI as an exposure-induced mediator-outcome confounder. The corresponding marginal controlled direct effect ORs are presented in Supplementary Table S1, available as Supplementary data at *IJE* online.

**Table 3:** Odds ratios and 95% confidence intervals for the total and the conditional controlled direct effects of Mediterranean diet on hip fracture with respect to T2DM as a mediator

	Total effect	Conditional controlled direct effect with respect to T2DM as a mediator		
	Model 1^a^	Model 2^b^	Model 3^c^	Model 4^d^
	OR (95% CI)	OR (95% CI)	OR (95% CI)	OR (95% CI)
0 (reference) (lowest adherence)	1.00	1.00	1.00	1.00
1	0.82 (0.71, 0.95)	0.82 (0.71, 0.95)	0.82 (0.71, 0.94)	0.82 (0.71, 0.95)
2 (highest adherence)	0.75 (0.62, 0.91)	0.75 (0.62, 0.91)	0.73 (0.61, 0.90)	0.73 (0.60, 0.88)

All models include the same set of confounders C: age, education, physical activity, smoking status, living alone status, calcium supplement use, vitamin D supplement use, total energy intake and Charlson comorbidity index.

OR, odds ratio; CI, confidence interval.;T2DM, type 2 diabetes mellitus; BMI, body mass index; MSM, marginal structural model.

a Estimated using logistic regression conditional on confounders C.

b Estimated using logistic regression conditional on confounders C and the mediator T2DM.

c Estimated using logistic regression conditional on confounders C, the mediator T2DM and the exposure-induced mediator-outcome confounder BMI.

d Estimated using a conditional marginal structural model with stabilized inverse probability weights for the mediator T2DM, conditional on confounders C. Please refer to Supplementary Table S1 for estimates for the marginal controlled direct effect based on a marginal structural model that is marginalized over the distribution of confounders (C).

To further separate the direct and mediating effects in the presence of potential sequential mediators, we applied flexible mediation analysis with multiple mediators (Table 4). The odds ratios for the natural direct effect (i.e. the result of mMED being changed with neither BMI nor T2DM being affected by this change) were 0.82 (0.71, 0.94) and 0.74 (0.61, 0.89) in the medium and highest level of adherence to mMED, respectively, compared with the lowest adherence category. The natural indirect effect and partial indirect ORs of approximately 1.00 indicate that, compared with the lowest level of mMED, changing BMI or T2DM status to what it would have been if they instead were at the intermediate level of mMED adherence would have no or minor effects on hip fracture.

**Table 4:** Odds ratios for the component effects from the natural effects model E{Y(a, M_1__*i*_(aʹ), M_2__*i*_(a′′, M_1__*i*_(aʹ)))|C} estimating the effect of Mediterranean diet (mMED) on hip fracture

mMED	Natural direct effect (a)^a^	Natural indirect effect (aʹ)^b^	Partial indirect effect (a′′)^c^
	mMED → hip fracture	mMED → BMI → hip fracture	mMED → T2DM → hip fracture
		mMED → BMI → T2DM → hip fracture	
Mediterranean diet score (mMED)	OR (95% CI)	OR (95% CI)	OR (95% CI)
0 (reference) (lowest adherence)	1.00	1.00	1.00
1	0.819 (0.710, 0.945)	1.006 (0.994, 1.017)	0.998 (0.989, 1.007)
2 (highest adherence)	0.737 (0.609, 0.893)	1.022 (1.004, 1.040)	0.989 (0.977, 1.002)

a The natural direct effect odds ratio corresponds to the effect of Mediterranean diet score (mMED) on risk of fracture through neither body mass index (BMI) (M_1_) nor type 2 diabetes mellitus (T2DM) (M_2_).

b The natural indirect effect odds ratio corresponds to the effect mediated by exposure-induced changes in body mass index (BMI) (M_1_), thus also including the path mMED → BMI → T2DM → hip fracture.

c The partial indirect effect odds ratio corresponds to the effect mediated solely by exposure-induced changes in type 2 diabetes mellitus (T2DM) (M_2_).

## Discussion

Based on our previous observation that greater adherence to a Mediterranean diet was associated with lower risk of hip fracture in this cohort,^6^ we aimed to investigate whether there is a direct effect of Mediterranean diet on hip fracture risk that is not mediated through T2DM. To overcome some of the potential biases that can arise when using traditional mediation analysis, we applied inverse probability weighting of marginal structural models. Due to the complex causal relations of diet, BMI, T2DM and hip fracture, we further explored the different mediating paths, treating BMI and T2DM as causally ordered mediators. Using different methods for effect estimation, we observed a direct effect of adherence to Mediterranean diet on hip fracture, not mediated by T2DM or BMI. The interpretation of the potential indirect effects is dependent on the estimation method used.

The traditional mediation method that compares the total effect of Mediterranean diet on hip fracture with the conditional direct effect not mediated by T2DM will be biased in our setting (Figure 1). Omitting BMI from the model (as in Model 2) results in residual confounding (BMI as an exposure-induced confounder of T2DM’s effect on hip fracture),^18^^,^^31^^,^^32^ and including both T2DM and BMI in the model (as in Model 3) will block the pathway mMED→BMI→hip fracture and thus bias the conditional controlled direct effect measure. Model 3, however, corresponds to the conditional controlled direct effect with respect to T2DM and BMI as joint mediators. The MSM conditional on confounders^18^ (Model 4) circumvents these biases by estimating the conditional controlled direct effect of mMED on hip fracture not going through T2DM, using inverse probability weighting where a weight is created for the mediator (T2DM). Controlled direct effect estimates marginalized over the confounders are presented in Supplementary Table S1. The MSM method is further advantageous to Models 2 and 3 in that it does not require the assumption of no unmeasured confounding^19^^,^^20^ between BMI and hip fracture. Although the effect measures presented within this paper are conditional on confounders, comparison of the total effect odds ratios with the controlled direct effect estimates are still hampered due to the non-collapsible nature of the odds ratio.^33^^,^^34^ Adding a true mediator to a regression model will lead to attenuated estimates for the exposure effect and, at the same time, adding a variable that is not a confounder or a mediator to a logistic regression model might lead to ORs further away from 1, due to non-collapsibility. Thus, if no change in estimate is seen after addition of a potential mediator to a logistic regression model, one may draw wrong conclusions regarding presence of mediation. Importantly, mediation effects will be underestimated when based on the difference-in-coefficients method using logistic regression,^34^ and we cannot exclude that they do not exist.

In contrast, the natural direct and indirect effects ORs can be compared with the total effect, since they are conditional on exposure levels and the same set of confounders; the product of the path-specific effects is approximately equal to the total effect. The natural direct and natural indirect effects of (mediated by BMI and T2DM as sequential mediators) suggest no or minor indirect effects in contrasting directions, which may be explained by counteracting effects in the complex biological pathway.

It is possible that the mediating effects of T2DM and BMI on hip fracture cancel each other out, so that it seems that there is little or no average mediating effect of the two. Potential mechanisms include that a higher adherence to a Mediterranean diet leads to lower BMI^12^ and therefore lower bone mineral density^35^ (associated with increased hip fracture risk), whereas the lower BMI may also lead to a lower incidence of T2DM and thereby lower risk of hip fracture.^36^ The estimated natural indirect effect explicitly combines these pathways, and cancelling out of effects could explain the—potentially by chance—small negative effect. The remaining partial indirect effect going through T2DM seems small. Thus, despite application of methods for the separation of mediating effects, our example illustrates that when complex causal relations exist, it may still not be possible to isolate the effect of main interest, in our case the natural indirect effect of mMED on hip fracture with T2DM as a mediator.

The interpretation of our results could be that the effect of mMED on hip fracture risk is primarily not mediated through T2DM, nor through BMI and T2DM, but rather through other pathways not examined in this study. Such pathways may include dietary constituents of the key food groups (plant foods, olive oil and fish) that have anti-inflammatory and anti-oxidant effects leading to bone and muscle sparing consequences and therefore fracture prevention.^37–44^

In the estimation of the conditional controlled direct effect (using IPW-MSM conditional on confounders) we fix the mediator to a certain level, whereas in the estimation of natural direct and indirect effects we fix the mediator to the natural value it would have been, given a certain level of exposure. Both methods rely on pre-specified assumptions. Even if the counterfactual framework allows for analysis and interpretation of mediation effects, critique of the methods includes that the combination of counterfactuals assessed in mediation analysis are constructs that can never be observed. Because controlled direct effects generally are closer to interventional scenarios where intermediates can be intervened upon (although interventional interpretations cannot always be made), they may be of greater interest in policy evaluation and planning.^45^ Natural direct and indirect effects are of greater interest in evaluating the mechanisms of action between and exposure and an outcome via any potential mediators.^46^

To our knowledge there has been no research into the potential mediating effects of diet on hip fracture. Previous research examining the mediated effects of Mediterranean diet on childhood obesity^47^ and Alzheimer’s disease^48^ used standard regression models, which may lead to biased estimates.^31^ Other mediation approaches have primarily focused on considering multiple mediators jointly^49^ and not decomposing effects into different mediating pathways.

Strengths of this study include the application of two recently developed mediation methods applied to a three-level categorical exposure, the large study population with a large number of hip fractures ascertained from official registers in a valid way with minimal loss to follow-up and the longitudinal design allowing temporal ordering of exposure, mediator and outcome variables, a prerequisite for mediation analysis. We were further able to take a large number of potential confounders into account, including comorbidity based on patient records. However, we cannot completely exclude the possibility of residual confounding, for instance by health seeking behaviour that is difficult to capture but can partly be accounted for by the adjustment for supplement use. The assumption of no residual confounding is further essential for causal inference.

Limitations include the self-reported nature of diet, height, weight, diabetes and covariates (Supplementary Discussion, available as Supplementary data at *IJE* online). Misreporting of or changes in dietary habits would lead to bias towards the null for the effect of Mediterranean diet on diabetes and hip fracture. However, adherence to dietary patterns has been shown to be fairly stable over a period of 8–10 years.^50^^,^^51^ Restricting the duration of T2DM to incident cases in 1997–2009 is an inherent limitation of the available data that may limit our power to detect possible mediating effects, since duration will likely influence fracture risk. This restriction may have introduced selection bias since we did not consider hip fractures and deaths occurring in 1997–2009. With repeat exposure assessments and exact dates of diabetes onset, we would have been able to apply recently developed methods to handle longitudinal mediation and time-to-event outcomes^52^^,^^53^ to overcome such limitations.

Our application of recently developed statistical techniques for counterfactual mediation analysis provides evidence for an effect of Mediterranean diet on hip fracture risk which is not mediated through T2DM. Although mediating effects of Mediterranean diet on hip fracture via T2DM (and BMI) seem minor, this may in fact be due to cancelling out of effects, and mediating effects may therefore be present. Still, our results suggest that the reduced risk of hip fracture with high adherence to a Mediterranean diet is likely to be largely mediated via the diet’s effect on other biological processes, which require further research.

Data are not freely available but it is possible to contact each cohort to request access.

## Supplementary data

Supplementary data are available at *IJE* online.

## Funding

This work was supported by grants from the Swedish Research Council: no. 2015–05997 and no. 2015–03527. We acknowledge SIMPLER [www.simpler4health.se] for provision of facilities and experimental support. SIMPLER receives funding through the Swedish Research Council under the grant no. 2017–00644.

## Supplementary Material

dyaa239_Supplementary_Data
