# Supplementary material for: Is the effect of Mediterranean diet on hip fracture mediated through type 2 diabetes mellitus and body mass index?
Source: Int J Epidemiol. 2020 Dec 25;50(1):234–44. doi: 10.1093/ije/dyaa239 (PMC7938512; doi:10.1093/ije/dyaa239)
Supplement: dyaa239_Supplementary_Data [file dyaa239_supplementary_data.docx]

**Supplementary information**

**Adam Mitchell et al. Is the Effect of Mediterranean diet on Hip Fracture Mediated through Type 2 Diabetes Mellitus and Body Mass Index?**

**Abbreviations**

BMI: body mass index; CDE: controlled direct effect; IPW: inverse probability weighting; IQR: inter quartile range; mMED: modified Mediterranean Diet Score; MSM: marginal structural model; NDE: natural direct effect; NIE: natural indirect effect; PIE: partial indirect effect; T2DM: type 2 diabetes mellitus

**Contents**

**Supplementary Figure S1**

**Supplementary Figure S2**

**Supplementary Figure S3**

**Supplementary Figure S4**

**Supplementary Methods**

Supplementary Table S1

Supplementary Table S2

**Supplementary Discussion**

**Supplementary References**


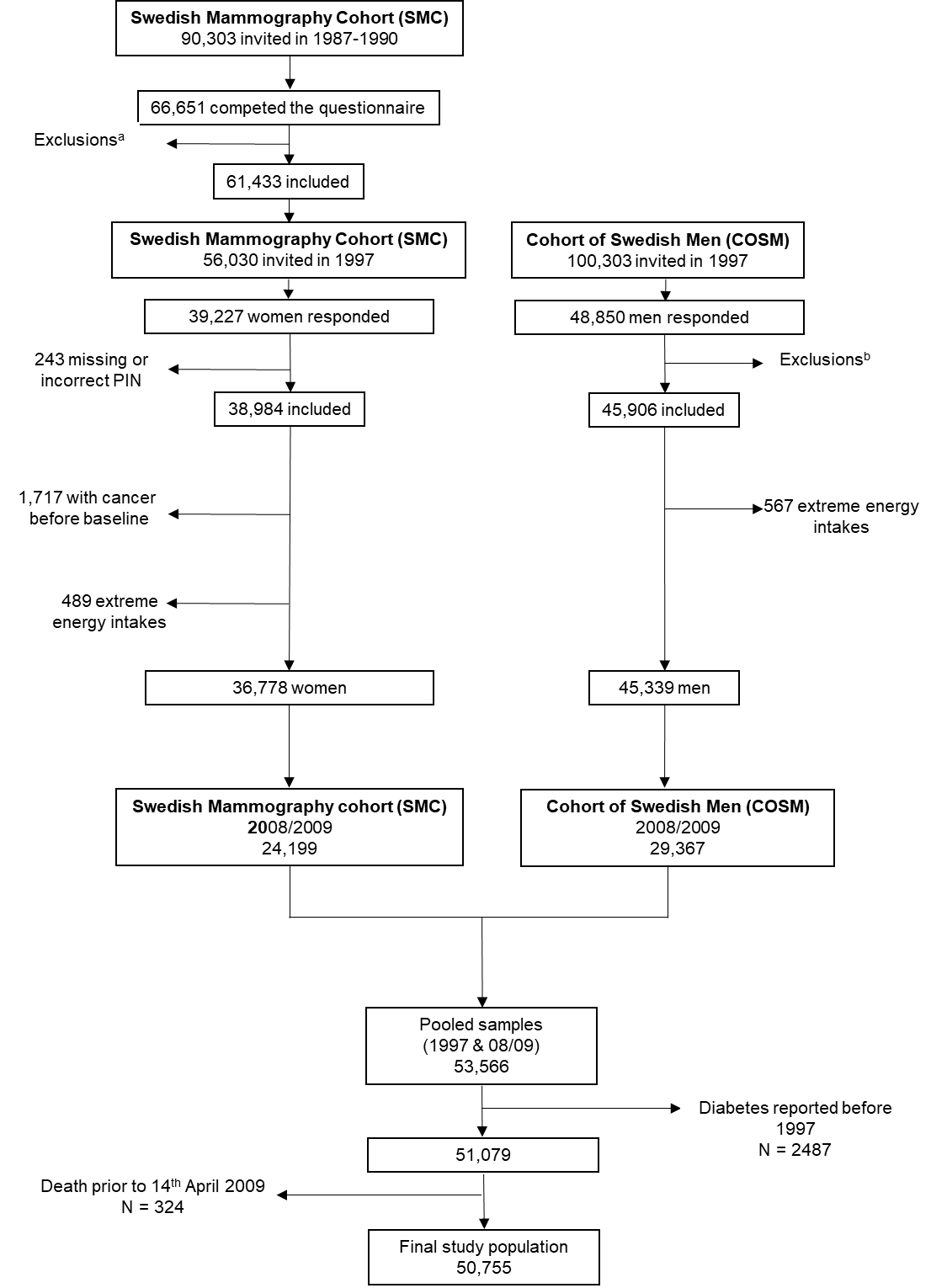


Death between 14^th^ April 2009 – 31^st^ December 2014 N = 5998

**Supplementary Figure S1.** Flow chart of the study cohort.

PIN (personal identification number).

^a^ Exclusions included those with missing or incorrect PIN, cancer diagnosis (except non-melanoma skin cancer) before baseline (in 1987-1990) and extreme energy intake (three standard deviations from the mean value for the log-transformed energy intake).

^b^ Exclusions included those with missing or incorrect PIN and cancer diagnosis (except non-melanoma skin cancer) before baseline.

**Supplementary Figure S2.** Cumulative incidence of hip fractures in women (dashed line) and men (solid line). 1386 hip fractures (849 women, 537 men).

**Supplementary Methods**

**Dietary assessment for calculation of the Mediterranean diet score**

We assessed habitual dietary intake over the previous year by a valid and reproducible 96-item food-frequency questionnaire (FFQ) in 1997 (1, 2). The eight possible frequency categories ranged from zero times/month to more than three times/day. FFQ responses were converted to average daily intakes based on age and sex-specific portion sizes from the Swedish National Food Agency database (3). Alcohol intake (ethanol, g/day) was calculated as the reported frequency of different alcoholic beverages times by ethanol concentration, multiplied with the reported amount consumed at each occasion (4).

**Assumed causal effects**

The association between Mediterranean diet and hip fracture was previously established in the two cohorts (5). Adherence to a Mediterranean diet is associated with a reduced risk of T2DM (6). The majority of studies have shown Mediterranean diet to be associated with lower BMI (7). Greater BMI however is associated with greater risk of T2DM (8). Within this study we estimated the association between T2DM at baseline (1997) and incident hip fracture (between 1998 and 2014) using Cox proportional hazards regression with censoring at time of death or end of follow up which revealed a greater risk of hip fracture in those with T2DM (HR 1.59 [1.45, 1.75]) compared to those without.

**Total effect**

The total effect of mMED (A) on hip fracture risk (Y):

$$\frac{E\left\{ Y\left( 1 \right) | C \right\} / (1-E\{Y(1)|C\})}{E\left\{ Y\left( 0 \right) | C \right\} / (1-E\{Y(0)|C\})}$$

was estimated using a logistic regression of mMED on hip fracture conditional on confounders C:

logit $\hat{P}$(*Y_i_*=1|*A_i_*=*a*, *C_i_*=*c*) = $\hat{\beta}$_0_ + $\hat{\beta}$_1_*A_i_* + $\hat{\beta}$_2_*C_i_* (Table 1, Model 1)

**Traditional methods for estimating the controlled direct effect with respect to T2DM as a mediator**

The controlled direct effect of mMED on hip fracture risk not mediated by T2DM

$$\frac{E\{Y(1, m) | C\} / (1-E\{Y(1, m) | C\})}{E\{Y(0, m) | C\} / (1-E\{Y(0, m) | C\})}$$

was estimated using two models.

First, we estimate this effect by adding T2DM (M) as a covariate to the model for estimation of the total effect, conditional on the set of confounders C:

logit $\hat{P}$(*Y_i_*=1|*A_i_*=*a*, *M_i_*=*m*, *C_i_*=*c*) = $\hat{\gamma}$_0_ + $\hat{\gamma}$_1_*A_i_* + $\hat{\gamma}$_2_*M_i_ +* $\hat{\gamma}$_3_*C_i_* (Table 1, Model 2)

However, exp($\hat{\gamma}$_1_) is not a valid estimator of the target estimand under the assumptions outlined in Figure 1. This is because conditioning on T2DM opens up the path mMED → T2DM ← BMI → hip fracture and the estimator may thus suffer from bias due to residual confounding from the exposure-induced mediator-outcome confounder BMI. A directed acyclic graph (DAG) illustrating the underlying causal assumptions needed for exp($\hat{\gamma}$_1_) in model 2 to be a valid estimator of the target estimand is shown in Supplementary Figure S3.

We thereafter included BMI, an exposure-induced confounder of the mediator outcome association (L), as a covariate in addition to T2DM and covariates C:

logit $\hat{P}$(*Y_i_*=1|*A_i_*=*a*, *M_i_*=*m*, *C_i_*=*c, L_i_=l*) = $\hat{\delta}$_0_ + $\hat{\delta}$_1_*A*_i_ + $\hat{\delta}$_2_*M_i_ +* $\hat{\delta}$_3_*C_i_* + $\hat{\delta}$_4_*L_i_* (Table 1, Model 3)

Again, exp($\hat{\delta}$*_1_*) is not a valid estimator under the assumptions outlined in Figure 1. This is because conditioning on BMI blocks one of the pathways of interest (mMED →BMI → hip fracture). Also, if there is unmeasured confounding (U) between BMI and hip fracture, this estimate may suffer from collider bias (by opening up the path mMED → BMI ←U → hip fracture). A DAG illustrating the underlying causal assumptions needed for exp($\hat{\delta}$*_1_*) to be a valid estimator of the target estimand using model 3 is shown in Supplementary Figure S4. However, note that exp($\hat{\delta}$*_1_*) would be a valid estimator of the controlled direct effect with respect to T2DM and BMI as a joint set of mediators under the assumptions outlined in Figure 1.


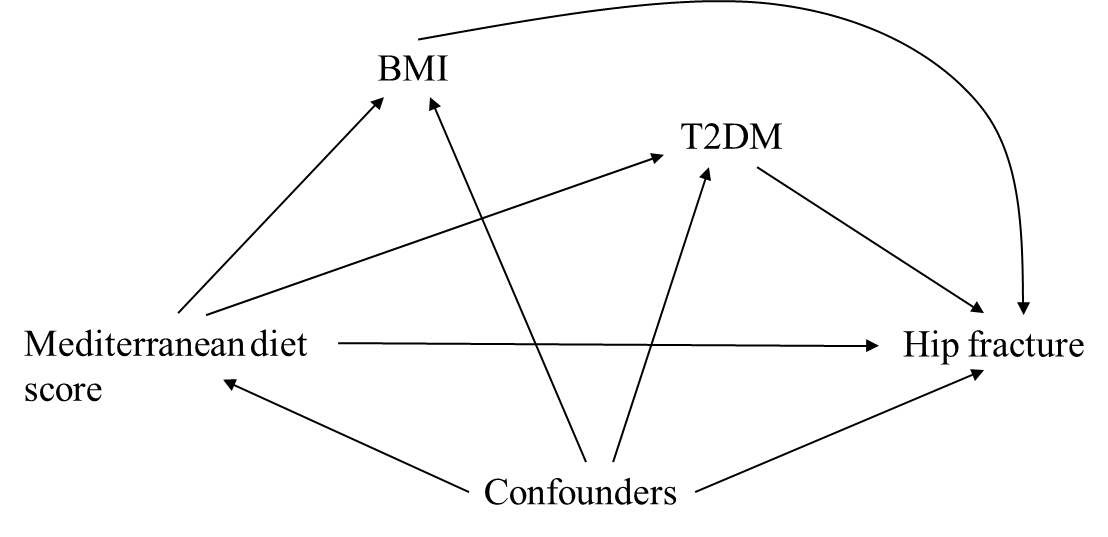


**Supplementary Figure S3.**

A hypothetical directed acyclic graph illustrating the underlying causal assumptions needed for exp($\hat{\gamma}$_1_) in model 2 to be a valid estimator of the controlled direct effect of effects of Mediterranean diet on fracture risk with respect to type 2 diabetes (T2DM) as a mediator and baseline confounders (C).


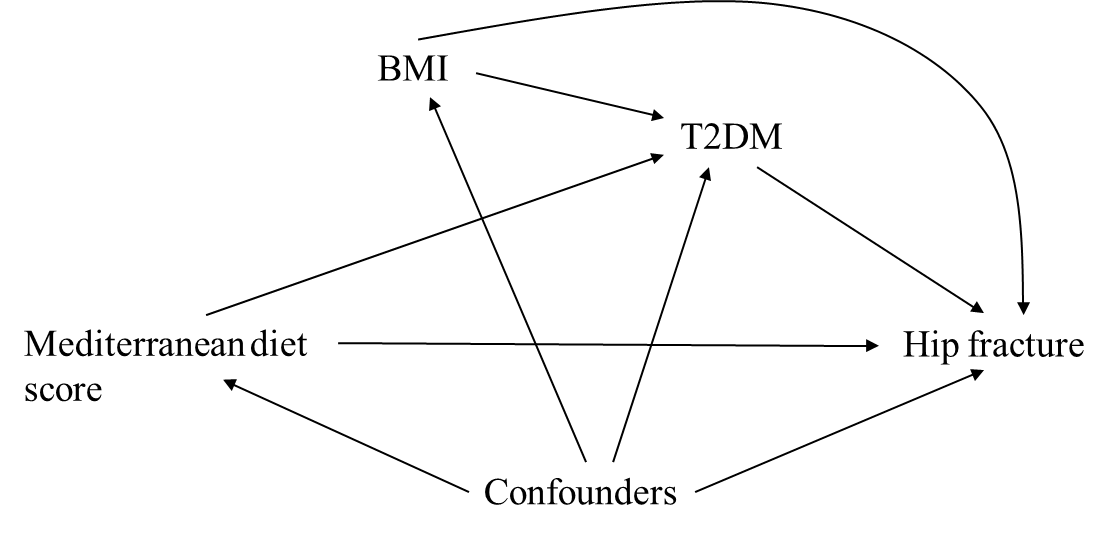


**Supplementary Figure S4.**

A hypothetical directed acyclic graph illustrating the underlying causal assumptions needed for exp($\hat{\delta}$*_1_*) in model 3 to be a valid estimator of the controlled direct effect of of Mediterranean diet on fracture risk with respect to type 2 diabetes (T2DM) as a mediator, BMI as common cause of T2DM and hip fracture, and baseline confounders (C).

**Inverse probability weighting (IPW) of marginal structural models (MSM) for estimating the controlled direct effect not mediated by T2DM**

Based on Figure 1 and assuming no unmeasured confounding, positivity and correct model specification, we estimated the controlled direct effect of mMED on the risk of hip fracture not mediated through T2DM using IPW and MSM (9, 10) (Table 1, Model 4), as detailed below, to avoid the biases highlighted above for models 2 and 3. The controlled direct effect of mMED (in 1997) on hip fracture (in 2009-2014)

$$\frac{E\{Y(1, m) | C\} / (1-E\{Y(1, m) | C\})}{E\{Y(0, m) | C\} / (1-E\{Y(0, m) | C\})}$$

when the mediator T2DM (in 2009) is set to a fixed level (m) estimates the effect of mMED on hip fracture not mediated through T2DM; that is, the effect of mMED on hip fracture after intervening so that the incidence of T2DM (in 1997-2009) is 0. This method eliminates the potential bias from the exposure-induced mediator outcome confounding present in models 2-3 and does not require the assumption of no unmeasured confounding between BMI and hip fracture.

We fitted two MSMs with inverse probability weights (IPW) as weighted logistic regression models with hip fracture as outcome, including the three-level mMED as exposure and T2DM as a binary mediator: one MSM that estimates marginal controlled direct effects (presented in Supplementary Table S1) and one MSM that estimates conditional controlled direct effects (model 4). For each individual in the study population, stabilised weights were generated for the exposure ($w_{A}$) and the mediator ($w_{M}$). Using ordered logistic regression, we calculated *w*_A_ as the probability of exposure (for the observed exposure level) divided by the conditional probability of exposure (for the observed exposure level), given observed confounders:

$$w_{A}=\frac{P(A_{i}=a)}{P(A_{i}=a|C_{i}=c)}$$

where a = the actual mMED category the individual had; c = the actual covariate values the individual had.

Using logistic regression, we calculated *w*_M_ as the probability of the mediator T2DM (M) given the exposure (A), divided by the probability of T2DM given the exposure, confounders (C), and the exposure-induced mediator-outcome confounder BMI (L):

$$w_{M}=\frac{P(M_{i}=m|A_{i}=a)}{P(M_{i}=m|A_{i}=a, C_{i}=c, L_{i}=l)}$$

where a = the actual mMED category the individual had, c = the actual covariate values the individual had, m = the actual mediator (T2DM) value each individual had, l = the actual exposure-induced mediator-outcome covariate (BMI) value each individual had.

Predicted probabilities for the numerator and denominator were assigned based on the category of actual mMED observed (9, 11).

We then took a product of these two weights for each individual:

$$w_{CDE}=w_{A}\times w_{M}$$

The distributions of the stabilised weights $w_{CDE}$ for each level of exposure were: (1) Mean=0.96, Range=0.04–54.77, IQR=0.54–1.09; (2) Mean=1.01, Range=0.08–14.14, IQR=0.91-1.03; and (3) Mean=0.98, Range=0.05–10.11, IQR=0.56-1.20.

Using the MSM approach to estimate the marginal controlled direct effect (CDE) on the odds ratio scale, we ran a logistic regression model of Y (hip fracture) on A (mMED) and M (T2DM):

logit $\hat{P}$*(Y_i_ =* 1*|A_i_*=*a*, *M_i_*=*m) =* $\hat{\eta}$_0_ + $\hat{\eta}$_1_*A_i_* + $\hat{\eta}$_2_*M_i_*

where each individual was weighted by $w_{CDE}$ (Supplementary Table S1). Robust standard errors using the sandwich estimator were calculated in each of the imputed datasets and Rubin’s rules were applied to calculate the estimates and 95% confidence intervals.

***Estimating the controlled direct effect not mediated by T2DM using a marginal structural model conditional on confounders***

The marginal structural model described above estimates effects marginalised over confounders (including the exposure-induced mediator-outcome confounder BMI for the mediator T2DM). The other models used in this paper estimates effects conditional on confounders. In order to make the estimates more comparable (please also refer to the section in the Discussion relating to non-collapsibility for comparison of odds ratios), we estimated the controlled direct effect using a marginal structural model conditional on confounders with stabilised inverse probability weights for the mediator T2DM (including the exposure-induced confounder BMI), conditional on confounders C (12) (Model 4, Table 1). We thus use weighting for the mediator ($w_{M}$ described above) and instead of using a weight for the exposure, we condition on the confounders C in the outcome model:

logit $\hat{P}$*(Y_i_ =* 1*|A_i_*=*a*, *M_i_*=*m, C_i_=c) =* $\hat{\eta}$_0_ + $\hat{\eta}$_1_*A_i_* + $\hat{\eta}$_2_*M_i_* + $\hat{\eta}$_3_*C_i_*

where each individual is weighted by $w_{M}$

The results from this analysis are presented in Table 3 (Model 4).

**Supplementary Table S1.** Marginal controlled direct effect of mMED on hip fracture not going through T2DM

| Mediterranean diet score (mMED) | OR (95% CI) |
| --- | --- |
| 0 (reference)  (lowest adherence) | 1.00 |
| 1 | 0.84 (0.72, 0.98) |
| 2  (highest adherence) | 0.63 (0.52, 0.78) |

Please, refer to Table 3, model 4, for the conditional controlled direct effect.

Confounders C include age, education, physical activity, smoking status, living alone status, calcium supplement use, vitamin D supplement use, total energy intake and Charlson comorbidity index.

**Flexible multiple mediator approach for estimation of natural direct and indirect effects**

To further separate the potential mediating paths, we applied a flexible analysis with multiple, causally ordered mediators (13) (Table 1, Model 5). This method allows effect decomposition into natural direct (NDE), natural indirect (NIE) and partial indirect effects given a specific set of mediators and when mediators are causally ordered. In this analysis and based on Figure 1, we considered BMI as a causally ordered mediator that precedes T2DM. We assume no unmeasured confounding for the effect of mMED on hip fracture, the effect of mediators (BMI, T2DM) on hip fracture conditional on exposure, or the effect of mMED on mediators (BMI, T2DM). We further assume that none of the mediator-outcome confounders are affected by mMED.

The mediator in natural effects estimation is not fixed to a certain level (as in estimation of the controlled direct effect) but rather takes on the level it would have naturally been under the counterfactual situation of mMED=a* (or a**). For estimation of natural effects in this setting with two sequential mediators (BMI (M_1_) → T2DM (M_2_)), we used nested counterfactuals to define the counterfactual outcome as Y(a,M_1_(aʹ),M_2_(aʺ,M_1_(aʹ))); that is the counterfactual outcome (hip fracture) that would be observed if mMED was set to a, and the mediators were set to the natural value they would have taken if mMED had been at the counterfactual level a*. This was then repeated for the second counterfactual (a**) of our three-level exposure mMED.

The estimates obtained from the natural effects model correspond to the NDE odds ratio of mMED on the risk of hip fracture through neither BMI nor T2DM (mMED → hip fracture), the NIE odds ratio mediated by exposure-induced changes in BMI (mMED → BMI → hip fracture and mMED → BMI → T2DM → hip fracture), and the partial indirect effect (PIE) odds ratio mediated solely by exposure-induced changes in T2DM (mMED → T2DM → hip fracture) (Table 2, Model 5).

Using logistic regression, we first calculated the predicted probability of T2DM (M_2_) conditional on exposure (A), BMI (M_1_) and confounders (C):

logit $\hat{P}$(*M*_2_*_i_*|*A_i,_ M_1i_, C_i_*) = $\hat{\zeta}$_0_ + $\hat{\zeta}$_1_*A_i_* + $\hat{\zeta}$_2_*M_1i_* + $\hat{\zeta}$*_3_A_i_M_1i_ +* $\hat{\zeta}$_4_C*_i_*

We then fitted a logistic regression model for the outcome (hip fracture) on the exposure (mMED), both mediators (BMI, T2DM) and covariates:

logit $\hat{P}$(*Y_i_* = 1|*A_i_*, *M*_1_*_i_*, *M*_2_*_i_*, *C_i_*) = $\hat{\zeta}$_0_ + $\hat{\zeta}$_1_*A_i_* + $\hat{\zeta}$_2_*M*_1_*_i_* + $\hat{\zeta}$_3_*M*_2_*_i_* + $\hat{\zeta}$_4_*A_i_M_1i_* + $\hat{\zeta}$_5_*A_i_M_2i_* + $\hat{\zeta}$_6_*M_1i_M_2i_* + $\hat{\zeta}$_7_*A_i_M_1i_M_2i_* + $\hat{\zeta}$*_8_C_i_*

We created three auxiliary variables (a, aʹ, aʺ) which correspond to the causal pathways we wish to decompose (natural direct effect, natural indirect effect and partial indirect effect), and extended the dataset by sequential replications as follows. Based on our three-level exposure and the choice to model the second mediator (13), we first create three copies of the dataset where a was set to the observed mMED level in the first copy, to the first counterfactual in the second copy, and to the second counterfactual in the third copy. Variables aʹ and aʺ were set to the observed value of mMED in each copy. Three copies of this first extended dataset were then created and aʺ was changed to the first counterfactual in the second copy and to the second counterfactual in the third copy. Each exposure level of mMED thus has two counterfactual levels. For each row of the final extended dataset, we computed weights as:

$$w_{NE}=\frac{\hat{P}(M_{2i}=m_{2}|A_{i}=aʺ,m_{1}, c)}{\hat{P}(M_{2i}=m_{2}|A_{i}=a,m_{1}, c)}$$

and used the fitted values from the outcome model ($\hat{E}(Y_{i}|A_{i}={aʹ,m}_{1},m_{2}, c)$) to impute nested counterfactuals Y*_i_*(a, M_1_*_i_*(aʹ), M_2_*_i_*(aʺ, M_1_*_i_*(aʹ))). The natural effects model E{Y(a,M_1i_ (aʹ), M_2i_(aʺM_1i_ (aʹ)))|C} was then fitted on the extended dataset using a weighted logistic regression model on the imputed counterfactuals given confounders on the auxiliary variables a, aʹ, and aʺ as:

logit $\hat{P}$*(Ê|a, aʹ, aʺ, C)* = $\hat{\theta}$_1_a + $\hat{\theta}$_2_aʹ + $\hat{\theta}$_3_aʺ *+* $\hat{\theta}$_4_*C*

where each observation was weighted by $w_{NE}$.

The estimate exp($\hat{\theta}$_1_) corresponds to the NDE odds ratio of mMED on the risk of hip fracture through neither BMI nor T2DM (a), exp($\hat{\theta}$_2_) to the NIE odds ratio mediated by exposure-induced changes in BMI (aʹ), and exp($\hat{\theta}$_3_)to the partial indirect effect (PIE) odds ratio mediated solely by exposure-induced changes in T2DM (aʺ).

Interaction terms between the auxiliary variables were not included in the main analyses since they were equal or very close to OR=1 as shown in Supplementary Table S2.

**Supplementary Table S2.** Odds ratios and 95% confidence intervals* for interaction terms included in the natural effect model

|  | a × aʹ | a × aʺ | aʹ × aʺ | a × aʹ × aʺ |
| --- | --- | --- | --- | --- |
| mMED medium vs. low | .9977 (.9955-1.0000) | 0.9997 (.9991-1.0002) | 1.0002 (1.0001-1.0003) | 1.0001 (1.0000-1.0002) |
| mMED high vs. low | .9970 (.9915-1.0024) | .9994 (.9974-1.0015) | 1.0022 (1.0018-1.0026) | 1.0000 (0.9998-1.0001) |

* 95% confidence intervals were calculated on the 20 imputed datasets but not bootstrapped (see below)

The calculation was performed in the 20 imputed datasets. The average of the point estimates (${\hat{\bar{\beta}}}_{MI}$) and the between-imputation variance of the point estimate ($\hat{V}$) were calculated. Further, the full calculation was performed with 1000 bootstrap samples in each of the 20 imputed datasets. The variance of the 1000 bootstrapped point estimates was extracted in each imputed dataset and the average of the 20 variances calculated ($\hat{W}$). The confidence limits for the point estimate was calculated using the invt() function in Stata as the point estimate (${\hat{\bar{\beta}}}_{MI}$) plus or minus:

$$invt\left( df, 0.975 \right)\cdot\sqrt{\hat{Var}\left( {\hat{\bar{\beta}}}_{MI} \right)}$$

where

$$df=19\cdot\left( 1+\frac{\hat{W}}{1.05\cdot\hat{V}} \right)$$

**Supplementary discussion**

Within this study we were able to apply two recently developed mediation methods to our data which included a three-level categorical exposure. For the causally ordered mediators model, each mMED level had two counterfactual levels.

Like all causal inference methods, the validity of our findings depends on our measures of exposure (mMED) and outcome (hip fracture) being accurate. We assume that BMI is sufficient to adjust for our mediator (T2DM) outcome (fracture) association and we also assume that our MSM was correctly specified with the correct and necessary baseline confounders.

We assume that our regression models and MSM were correctly specified, and that the consistency assumption holds (14), which states that the exposure is defined with enough specificity that different variants of the exposure do not have different effects on the outcome (15). We also assume positivity for all models and acknowledge that inverse probability weighting of marginal structural models require this assumption so that the probabilities in the denominator of the weights are nonzero (16). It is also important to note that although we adjusted our estimates for potential confounders, residual confounding may still exist (17). Alternatively, the measured confounders we did include may be measured with error for example physical activity and smoking status.

Dietary assessment is, like all measurements, prone to errors (18), however we used a valid and reproducible FFQ. Overweight and obese individuals may be more likely to under-report their energy intake and over-report intakes of healthy foods (19, 20), which may lead to potential misclassification of adherence to Mediterranean diet. Self-reported diabetes when compared to medical record review has a high positive predictive value of prevalent (91.8%) and incident diabetes (82.2%) and a high negative predictive value (94.5%) (21). Adjusting for potential reporting errors in BMI by comparing self-reported height and weight with measured height and weight in participants in the U.S. National Health and Nutrition Evaluation Survey (NHANES) did not greatly affect the estimates of BMI and increased all-cause mortality (22).

**Supplementary References**

1. Messerer M, Johansson SE, Wolk A. The validity of questionnaire-based micronutrient intake estimates is increased by including dietary supplement use in Swedish men. J Nutr. 2004;134(7):1800-5.

2. Rautiainen S, Serafini M, Morgenstern R, Prior RL, Wolk A. The validity and reproducibility of food-frequency questionnaire-based total antioxidant capacity estimates in Swedish women. Am J Clin Nutr. 2008;87(5):1247-53.

3. Bergstrom L, Kylberg E, Hagman U, Erikson H, Bruce A. The food composition database KOST: the National Administration’s information system for nutritive values of food. Swedish Vår Föda. 1991;43:439-47

4. Stackelberg O, Bjorck M, Larsson SC, Orsini N, Wolk A. Alcohol consumption, specific alcoholic beverages, and abdominal aortic aneurysm. Circulation. 2014;130(8):646-52.

5. Byberg L, Bellavia A, Larsson SC, Orsini N, Wolk A, Michaëlsson K. Mediterranean Diet and Hip Fracture in Swedish Men and Women. J Bone Miner Res. 2016;31(12):2098-105.

6. Koloverou E, Esposito K, Giugliano D, Panagiotakos D. The effect of Mediterranean diet on the development of type 2 diabetes mellitus: a meta-analysis of 10 prospective studies and 136,846 participants. Metabolism. 2014;63(7):903-11.

7. Buckland G, Bach A, Serra-Majem L. Obesity and the Mediterranean diet: a systematic review of observational and intervention studies. Obes Rev. 2008;9(6):582-93.

8. Schienkiewitz A, Schulze MB, Hoffmann K, Kroke A, Boeing H. Body mass index history and risk of type 2 diabetes: results from the European Prospective Investigation into Cancer and Nutrition (EPIC)-Potsdam Study. Am J Clin Nutr. 2006;84(2):427-33.

9. Nandi A, Glymour MM, Kawachi I, VanderWeele TJ. Using marginal structural models to estimate the direct effect of adverse childhood social conditions on onset of heart disease, diabetes, and stroke. Epidemiology. 2012;23(2):223-32.

10. VanderWeele TJ. Explanation in Causal Inference Oxford University Press; 2015. p. 126-34.

11. Robins JM, Hernan MA, Brumback B. Marginal structural models and causal inference in epidemiology. Epidemiology. 2000;11(5):550-60.

12. VanderWeele TJ. Marginal structural models for the estimation of direct and indirect effects. Epidemiology. 2009;20(1):18-26.

13. Steen J, Loeys T, Moerkerke B, Vansteelandt S. Flexible Mediation Analysis With Multiple Mediators. Am J Epidemiol. 2017;186(2):184-93.

14. VanderWeele TJ. Concerning the consistency assumption in causal inference. Epidemiology. 2009;20(6):880-3.

15. Rehkopf DH, Glymour MM, Osypuk TL. The Consistency Assumption for Causal Inference in Social Epidemiology: When a Rose is Not a Rose. Current epidemiology reports. 2016;3(1):63-71.

16. Cole SR, Hernan MA. Constructing inverse probability weights for marginal structural models. Am J Epidemiol. 2008;168(6):656-64.

17. Fewell Z, Davey Smith G, Sterne JA. The impact of residual and unmeasured confounding in epidemiologic studies: a simulation study. Am J Epidemiol. 2007;166(6):646-55.

18. Shim J-S, Oh K, Kim HC. Dietary assessment methods in epidemiologic studies. Epidemiol Health. 2014;36:e2014009.

19. Heitmann BL, Lissner L. Dietary underreporting by obese individuals--is it specific or non-specific? BMJ. 1995;311(7011):986-9.

20. Salle A, Ryan M, Ritz P. Underreporting of food intake in obese diabetic and nondiabetic patients. Diabetes Care. 2006;29(12):2726-7.

21. Jackson JM, DeFor TA, Crain AL, Kerby TJ, Strayer LS, Lewis CE, et al. Validity of diabetes self-reports in the Women's Health Initiative. Menopause (New York, NY). 2014;21(8):861-8.

22. Berrington de Gonzalez A, Hartge P, Cerhan JR, Flint AJ, Hannan L, MacInnis RJ, et al. Body-Mass Index and Mortality among 1.46 Million White Adults. N Engl J Med. 2010;363(23):2211-9.
